# Draft Genome Sequence of Thermophilic *Geobacillus* sp. Strain Sah69, Isolated from Saharan Soil, Southeast Algeria

**DOI:** 10.1128/genomeA.01447-15

**Published:** 2015-12-17

**Authors:** Oliver K. I. Bezuidt, Thulani P. Makhalanyane, Mohamed A. Gomri, Karima Kharroub, Don A. Cowan

**Affiliations:** a Centre for Microbial Ecology and Genomics, Department of Genetics, University of Pretoria, Pretoria, South Africa; b Equipe Métabolites des Extrêmophiles, Laboratoire de Recherche Biotechnologie et Qualité des Aliments, INATAA, Université Frères Mentouri, Constantine, Algeria

## Abstract

*Geobacillus* spp. are potential sources of novel enzymes, such as those involved in the degradation of recalcitrant polymers. Here, we report a *Geobacillus* genome that may help reveal genomic differences between this strain and publicly available representatives of the same genus from diverse niches.

## GENOME ANNOUNCEMENT

Thermophilic environments such as desert soils contain a diverse consortia of microbial taxa, which span both archaeal and bacterial lineages (1, 2). Efforts have focused on isolation of thermophilic archaea, principally due to their potential biotechnological applications (3). However, more recently, the need to identify novel thermostable hydrolytic enzymes has led to renewed interest in isolating *Geobacillus* spp., due to their capacity to degrade complex carbohydrates for application in bioethanol production (4). We sequenced the genome of a novel isolate from a hot Saharan soil in order to further understand the metabolic capacity of this genus. *Geobacillus* spp. are thermophilic, Gram-positive, spore-forming aerobic bacteria, many of which have demonstrated broad-specificity, carbohydrate-degradative traits (5).

*Geobacillus* sp. strain Sah69 was grown on nutrient agar and incubated at 55°C for 24 h. Genomic DNA was obtained from the isolate using a modification of the method first described by Miller and colleagues (6). The 16S rRNA gene was amplified and sequenced, confirming that isolate Sah69 belongs to the genus *Geobacillus*. Genomic DNA was sent for sequencing on an Illumina MiSeq DNA at MR DNA, Shallowater, TX, USA. The library preparation and sequencing was done as described elsewhere (7). The genome was assembled using DNAStar SeqMan NGen software, and annotated using the Rapid Annotation using Subsystems Technology (RAST) server (http://rast.nmpdr.org) (8). The draft genome had a total of 73 contigs with protein-encoding genes, 2,998,191 nucleotides, and a G+C content of 52.58%. RAST annotation revealed 102 RNAs and 3,372 coding sequences. One intact and one incomplete phage genome was predicted using the PHAST (Phage Search Tool) server (9). The genome was shown to possess the CRISPR-Cas (clustered regularly interspaced short palindromic repeats, CRISPR-associated) elements of the Csn1, Cas1, and Cas2 families (10). These elements are essential for the acquisition of resistance by bacteria against foreign genetic elements through the excision and integration of a genome fragment from invading DNA into its CRISPR arrays.

These data will help provide knowledge about the mechanisms behind microorganisms that thrive in extreme environments.

### Nucleotide sequence accession numbers.

This whole-genome shotgun project has been deposited at DDBJ/EMBL/GenBank under the accession number LLKS00000000. The version described in this paper is the first version, LLKS01000000.
